# Women Entrepreneurship: A Systematic Review to Outline the Boundaries of Scientific Literature

**DOI:** 10.3389/fpsyg.2020.01557

**Published:** 2020-07-17

**Authors:** Giuseppina Maria Cardella, Brizeida Raquel Hernández-Sánchez, José Carlos Sánchez-García

**Affiliations:** Department of Social Psychology and Anthropology, University of Salamanca, Salamanca, Spain

**Keywords:** entrepreneurship, women, literature review, gender gap, female entrepreneurship, barriers, work-family balance, economic development

## Abstract

In recent years, the study of women entrepreneurship has experienced great growth, gaining a broad consensus among academics and contributing above all to understanding all those factors that explain the difficulty of women in undertaking an entrepreneurial career. This document tries to contribute to the field of study, thanks to a systematic analysis through the publications present in the topic. For this purpose, 2,848 peer-reviewed articles were analyzed, published between 1950 and 2019, using the Scopus database (SCImago Research Group). Through the use of a series of bibliometric indicators it was possible to define the current state of research on the international scene. The analysis revealed that it is a multidisciplinary field of study and that has started to expand further since 2006, culminating in 2019, which makes it become a current and valid object of study. The analysis of the clusters allowed to isolate 6 different lines of research in which emerged, on the one hand, the importance of entrepreneurial education, social entrepreneurship and the socio-cultural context of reference (e.g., culture, family, and institutional support) as tools to overcome the gender gap, on the other, the importance that women entrepreneurship assumes in the economic growth of the country (especially in developing economies), promoting social inclusion and combating poverty and discrimination. The study presents an important contribution to reflect on current policies and to outline future lines of investigation.

## Introduction

Female entrepreneurs represent the fastest growing category of entrepreneurship worldwide and have received, especially in recent years, the attention of many academics. According to the emerging literature, women can make a significant contribution to entrepreneurial activity (Noguera et al., 2013) and economic development (Kelley et al., 2017; Hechevarría et al., 2019) in terms of creating new jobs and increasing the gross domestic product (GDP) (Bahmani-Oskooee et al., 2013; Ayogu and Agu, 2015), with positive impacts on reducing poverty and social exclusion (Langowitz and Minniti, 2007; Rae, 2015). The percentage of women who decide to pursue an entrepreneurial career is, however, lower than that of men (Elam et al., 2019), and this difference is greater as the level of development of the country increases (Coduras and Autio, 2013).

A theoretical framework used to explain this difference underscores the importance of economic and regulatory conditions (Estrin and Mickiewicz, 2011). For example, in the literature it is possible to trace substantially two macro-categories that have a different impact on the entrepreneurial activity of men and women. The first refers to the role of property rights underlying an entrepreneurial productive activity. In general, property rights facilitate access to resources and, in many institutional contexts, women are particularly limited in their access to the economic resources necessary for entrepreneurship (Brush et al., 2009), as entrepreneurs have to rely more on informal networks that usually tend to be dominated by men (Aidis et al., 2008). Furthermore, because of gender-defined social positioning, men can also be more effective in dealing with government officials (Bardasi et al., 2011).

The second focuses on a group of government-determined regulations and policies, such as welfare and system taxes. Some studies (Parker, 2009; Aidis et al., 2010) have found that a larger state sector militates against entrepreneurial activity. Therefore, tax and social security provisions can influence entrepreneurial entry through their direct impact on expected returns from entrepreneurial activities and opportunity costs. High levels and rising marginal tax rates can weaken incentives for opportunity-oriented entrepreneurs by reducing the potential, while higher levels of social assistance provide alternative sources of income and, therefore, by increasing alternative wages, they can reduce incentives for entrepreneurship. This appears particularly important in the case of women as a large state sector is dedicated to women offering security, educational services, health care and housing, but inevitably reducing their premiums.

However, among countries with similar economic conditions (Minniti, 2010; Dheer et al., 2019), this difference continues to exist between men and women when it comes to starting a business, which has led to calls to further expand the scope of explanatory factors (McGowan et al., 2015).

In line with this reasoning, there is empirical evidence that a woman's decision to start a business depends on her socio-cultural background (Ahl, 2006).

A first theme of analysis useful to explain the gap between men and women in entrepreneurship can be represented by the social roles and stereotypes that are culturally assigned to men and women.

The term gender was first introduced by Stoller to describe people based on biological physical characteristics, this would determine the individual's behavior. Based on these characteristics, men are expected to behave well masculine while women should think and to behave feminine. According to the social role theory (Eagly, 1987), gender stereotypes can make a person socially acceptable. When a role is associated with men, women they are not suited to the role because they do not have the necessary skills. The behavioral differences related to gender specific perceptions and preferences could explain the different inclination of men and women toward entrepreneurship. Koellinger et al. (2011) conducted an analysis in 17 countries showing a lower entrepreneurial propensity for women. In addition, the authors provided empirical evidence of gender differences related to self-efficacy and fear of failure.

Literature shows that entrepreneurs are described as aggressive and with high-risk proclivities (Bird and Brush, 2002), as well seem more socially inclined to achieve and obtain economic benefits, an image which does not fit in women (Ahl, 2004; Dileo and Pereiro, 2019), who seem closer to care and the emotional sphere, therefore, in pursuit of social value (Hechevarría et al., 2012; Urbano Pulido et al., 2014).

Additionally, in an analysis aimed to investigate how academics contribute to the perpetuation of stereotypes about female entrepreneurship, Ahl (2004) found that in all the texts reviewed, women entrepreneurs were considered secondary to men. The reasons for this “negative representation” remains the subject of international debate, for which there are no common results.

This stereotyped and male-centered vision discourages some women from participating in business activities, which could also have a consequence on people who interact with women at the community level, creating an additional barrier (Langowitz and Morgan, 2003). The results of the systematic analysis conducted by Sullivan and Meek (2012) suggested that the attributions of society and the different socialization processes relating to men and women may create obstacles for women due to the unequal distribution of assets and services, educational objectives and daily life activity expectations.

According to a study by Guzman and Kacperczyk (2019), females are 63% less likely than males to obtain external financing in terms of risk capital, and the most significant part of the gap derives from differences in gender.

The social construction of the entrepreneur as an independent and stereotyped man calls into question a second theme of analysis that can be limiting for women, namely the responsibility that women seem to have on the family/work issue (Jennings and Brush, 2013; Neneh, 2018). Boz et al. (2016) discovered that women who care most about the family have negative behaviors at work, consequently, the balance between family and work is more difficult for women entrepreneurs, which represents a fundamental obstacle to the growth of their businesses.

Other empirical evidence has shown the opposite. According to Thébaud (2015), work-family conflict can be an important factor that motivates women to start a business. For example, business creation can offer women considerable flexibility in terms of work hours (for example, work only a few hours a week or work at home) allowing them to find a balance between work and family commitments (Kirkwood and Tootell, 2008).

In this sense, the study by Rembulan et al. (2016), which analyzed differences in the work-family conflict between women who work as employees (98 employees) and those who work as entrepreneurs (91 entrepreneurs), showed that most female entrepreneurs have very low conflict in all aspects: time, tension, and behavior; unlike women who work as employees who tend to have higher conflict. One possible explanation may be in the gap of the annual income received. Specifically, the higher the income, the less the stress caused by the work- family conflict.

The literature has paid little attention to the analysis of women's motivations and expectations about entrepreneurship and how it really offers a better “balance” between family and work. McGowan et al. (2012) conducted a qualitative study with 14 women from Northern Ireland while they established and managed heir businesses, balancing family needs. The results showed that the motivation to engage in venturing was the desire to balance family responsibilities thanks to the greater flexibility that characterizes this type of work, with the desire to achieve personal independence.

However, entrepreneurship offered a partial answer. In some cases it has acted as a trigger for women to take a positive step they have been contemplating for some time. On the other hand, the negative realities of the company posed serious challenges to these women. For most of them, obtaining and maintaining an adequate balance between the domestic and working spheres of their lives has remained a constant challenge, a source of stress.

In addition, men and women cannot participate in the same entrepreneurial activity due to differences in the access to diverse forms of capital. For example, Johansen (2013) points out as issues the difficulty in obtaining support (institutional, family, and financial), fear of failure, self-assessment of the gender gap, and unfavorable social perceptions. Noguera et al. (2013) highlight fear of failure and self-efficacy as important barriers that hinder the propensity of women to pursue a business career. Other authors have reached similar conclusions in recent years (Wieland et al., 2019).

The results are not uniform, but despite the differences, these studies generally show that women entrepreneurs experience a greater lack of support than men when they try to access business resources (Langowitz and Minniti, 2007). However, the results of a study by Centindamar et al. (2012) on the relative importance of the three types of capital for business: human, family, and financial, underlined that, regardless of sex, these three types of capital influence the likelihood of becoming entrepreneurs. In addition, contrary to expectations, the impact of human capital on the probability of becoming an entrepreneur is greater for women than for men. The data also revealed that family capital facilitates the entry of women into entrepreneurship only in large families. No gender differences were observed with respect to the impact of financial capital.

From this outlined literature, it seems necessary to clarify the existing theoretical concepts to better explain the uniqueness of female entrepreneurship as an independent research topic. Over the years, in fact, the lack of specific research on the phenomenon (De Bruin et al., 2007), and a stereotypically male business model considered as a natural way for doing business (Bruni et al., 2004), caused a delay and underestimation of the study of women in the business process as an important area of research until the late 90's (Jennings and Brush, 2013). As Brush (1992) noted: “Women business owners are similar to males across some basic demographic factors, problems, and business characteristics, but they differ widely from male business owners across individual dimension related to education, work experience, skills, approach to venture creation / acquisition, business goals, problems and performance” (p. 24).

Global statistics also highlight this aspect. Although over the years there has been a significant increase in the number of women who have developed or undertaken an entrepreneurial activity, it will take at least another 108 years to completely close the gender between men and women, and 202 years to achieve equality between the two genders in the workplace. This is confirmed by the Global Gender Gap Report 2018 published by the World Economic Forum (2018), which taking into account four indicators: economic opportunity, political growth, training, health, and survival, showed in 2018 a 68% gap. The wage gap is almost 51%, and in 2018 women in leadership positions were only 34%. The same is also true for 2020 (Global Gender Gap score stands at 68,6%) (World Economic Forum, 2020).

In addition, according to the Global Entrepreneurship Monitor (GEM) 2018/2019, which provides an overview of the status of female entrepreneurship in 49 countries, Slovenia, Greece, Sweden, Switzerland, United Kingdom, and Turkey are the countries where women startups are less than half of men's. In some countries in Europe and North America, the levels of the TEA (Total Entrepreneurial Activity) rate for women do not reach 5% (Bosma and Kelley, 2019).

In startups around the world the situation is no different. As highlighted by the Startup Outlook 2018 survey, published by Silicon Valley Bank (SVB) (2019), 71% of new American companies do not have women on their board, and 57% do not have the top positions in the so-called C-Suite. Other information obtained from the new companies registered in CrunchBase indirectly confirms the data of the Svb Survey: in 2017, only 17% of the young innovative companies had a female co-founder.

This considered, it seems important to increase the percentage of women in entrepreneurship, an issue that has aroused political interest in recent years by emphasizing the possible economic benefits that could be derived from it (Carter et al., 2015), stating gender equality contributes to economic growth. In fact, the “global gender gap” is at the base of EU policy, as it identifies a clear economic logic to encourage women to become independent entrepreneurs (Carter et al., 2015; Sorgner et al., 2017). Nonetheless, this purely economic emphasis for multiple female entrepreneurs has been defined as an intention to “sell neoliberal values to defenders of gender equality” (Elomäki, 2015). Additionally, according to Boyd (2016), what is missing is a critical counterweight in the public debate: there is no collective questioning among all the actors who work from different fields to understand the gender-related discrimination, something necessary for a truly sustainable development.

This systematic analysis attempts to present an overview of the topic, tracing the current trend of research on women's entrepreneurship, highlighting the future directions of research, with the aim of deepening our understanding of this research branch.

Specifically, this article has two main objectives. The first is to highlight the growth of female entrepreneurship in scientific literature through the chronological distribution of publications and the productivity of authors, journals and countries. The second objective is to track the lines of research most developed and analyzed by the scientific community.

The article is organized in the following manner: first, we discuss the research review approach used in the article and, second, present the results of the analysis performed. Lastly, we present the conclusions that can be drawn from our analysis, the limitations of the study, and indications for future research.

## Materials and Methods

We carry out this systematic analysis of the literature to contribute to the systematization of scientific production on the relationship between entrepreneurship and women. In this sense, we have used the Scopus database, widely recognized in the scientific community, with more than 27 million abstracts, and is currently considered the largest database of scientific literature (Burnham, 2006).

The selected search terms included the words “entrepren^*^” and “women,” using the “AND” Boolean connector and including “all fields” as a search field, with no time margins. The bibliographic search ended in December 2019, generating a total of 4,164 documents published between 1950 and 2019.

The final selection of the articles was made using the following inclusion criteria: (i) scientific articles published in peer-reviewed journals, since they are considered valid sources of knowledge (Podsakoff et al., 2005), (ii) written in English. All articles related to the year 2020, articles written in a language other than English, conference presentations, book and thesis chapters, etc. have been removed. Although this may represent a limitation since part of scientific contributions has been excluded, we believe it is an effective way to garantee the quality of the work thanks to their reliability in the academic world and the rigorous review processes that are usually carried out (Nicholas et al., 2015).

**Figure 1:**
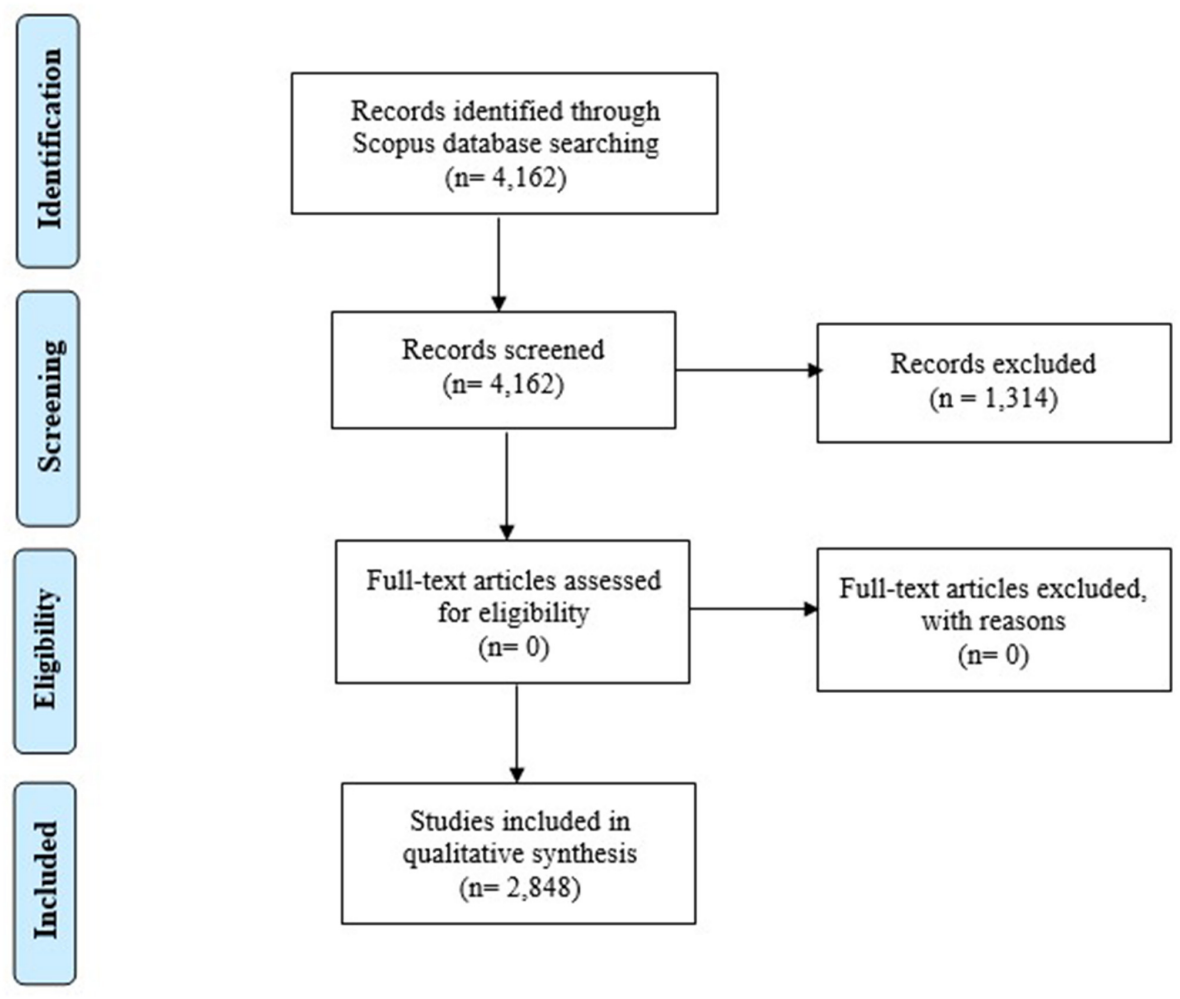
Flow Diagram—PRISMA method, 2009.

This selection phase narrowed the field, producing the final result of 2,848 scientific articles. To minimize the subjective component and possible attribution errors, we followed the guidelines of the PRISMA method (Liberati et al., 2009; Moher et al., 2009; Urrútia and Bonfill, 2010). This allows replicating the work (Lourenço and Jones, 2006; Pittaway and Cope, 2007), and we used a series of bibliometric indicators to analyze the temporal evolution of scientific production, the most influential authors on the subject, the most productive scientific journals with regards to the number of articles published, and countries with the highest number of scientific contributions. Figure 1 shows the flow chart of the bibliographic research according to the recommendations of the PRISMA method.

The analysis was carried out using descriptive statistics to describe the general panorama of female entrepreneurship. In addition, VOSviewer software version 1.6.10 (Van Eck and Waltman, 2010, 2014) was used, a bibliometric technique that allows the graphic representation, identification and classification of groups in an associated strategic matrix based on similarities and differences (distance based mapping). Although the qualitative analysis of the literature may be influenced by the subjectivity of the author, this method solves this problem. Using the keywords used by the authors themselves, it allows to reduce the distortion deriving from subjective variables, moreover, the graphic creation of maps allows to examine the deep relationships between the variables, which helps to better understand the nature of a research field, becoming an indisputable analysis tool (Vallaster et al., 2019), currently used (Martínez-López et al., 2018).

Specifically, an citation analysis was conducted to identify great impact of authors and co-citation analysis was conducted in order to measure the similarity between authors, journals and countries. Keyword co-ocurrence analysis was used to analyze the type and strength of the relationship between different fields of science. In Table 1 we report the first 5 keywords that in our study had greater strength.

**Table 1:** Occurrence of most relevant keywords.

**Rank**	**Keywords**	**Occurrence**	**Link strenght**
1	Gender	536	1388
2	Entrepreneurship	535	1203
3	Women	248	951
4	Women Entrepreneurs	172	409
5	Female Entrepreneurship	64	172

*Elaborated by the Authors. Source: VOSviewer 1.6.10*.

## Results and Discussion

### Bibliometric Analysis

**Figure 2:**
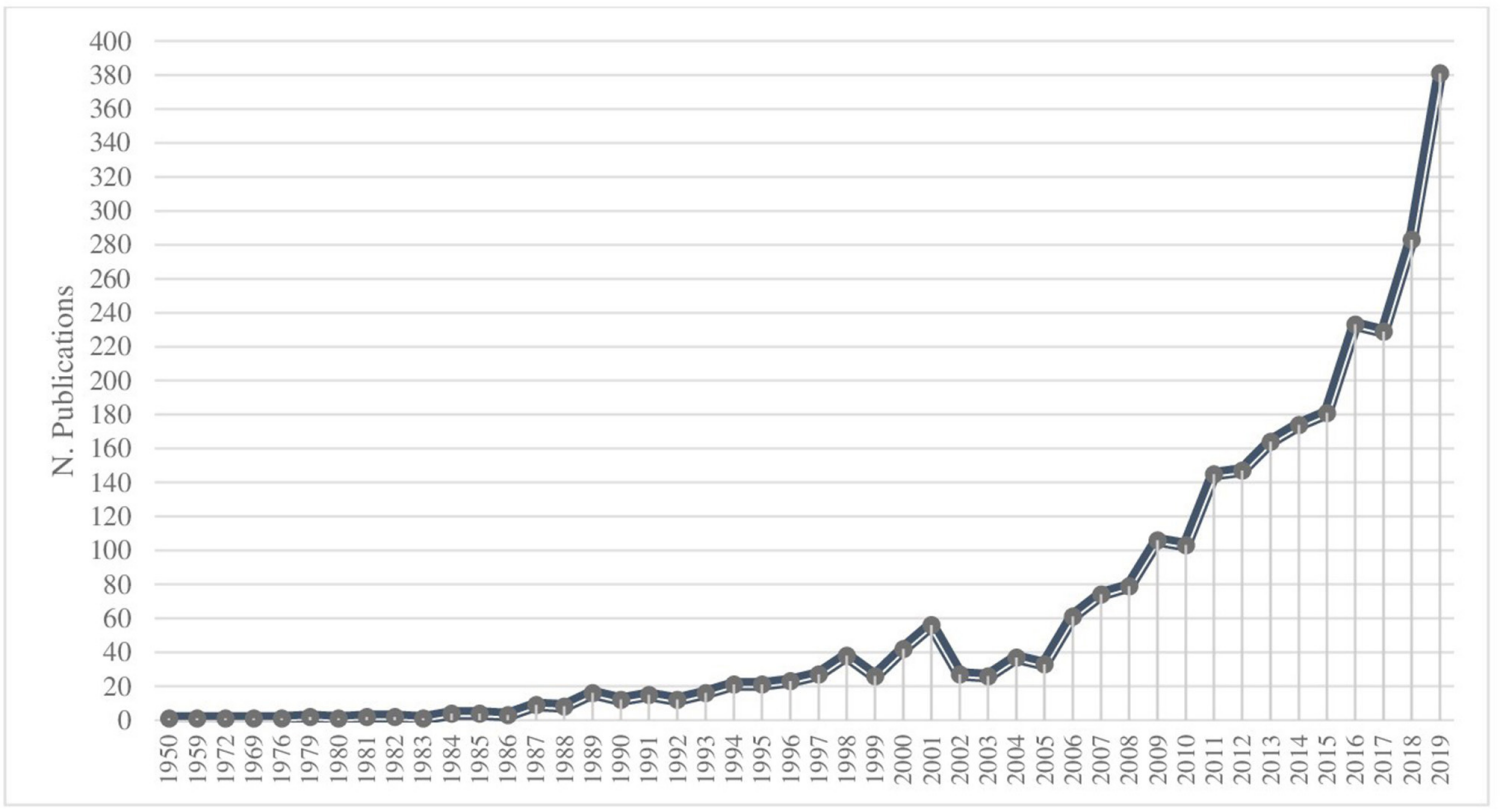
Evaluation of scientific publications per years. Source: Elaborated by the Authors.

Figure 2 shows the progress of scientific research on entrepreneurship and women over the years. It is a research field that, although studied for 70 years (the first article dates back to 1950), has developed mainly in recent years, registering a significant increase since 2006 (*n* = 61) and reaching the highest peak of publications in 2019 (*n* = 381). This increase could suggest a change in interest in scientific research and a continuous and growing evolution of research in the field of female entrepreneurship as a valid trend.

To identify the “research front” on female entrepreneurship through temporal overlap, we used the analysis of the co-ocurrence of keywords (with a minimum of five keywords).

The “research front” (Price, 1965) is the growing tip of literature and characterize the transient nature of a research field. It is a dynamic analysis, as it is affected by changes in the research area, as well as by the importance, over the years, of a specific research linea. The identification of the research front helps scholars to outline the most current trends in literature (Boyack and Klavans, 2010).

**Figure 3:**
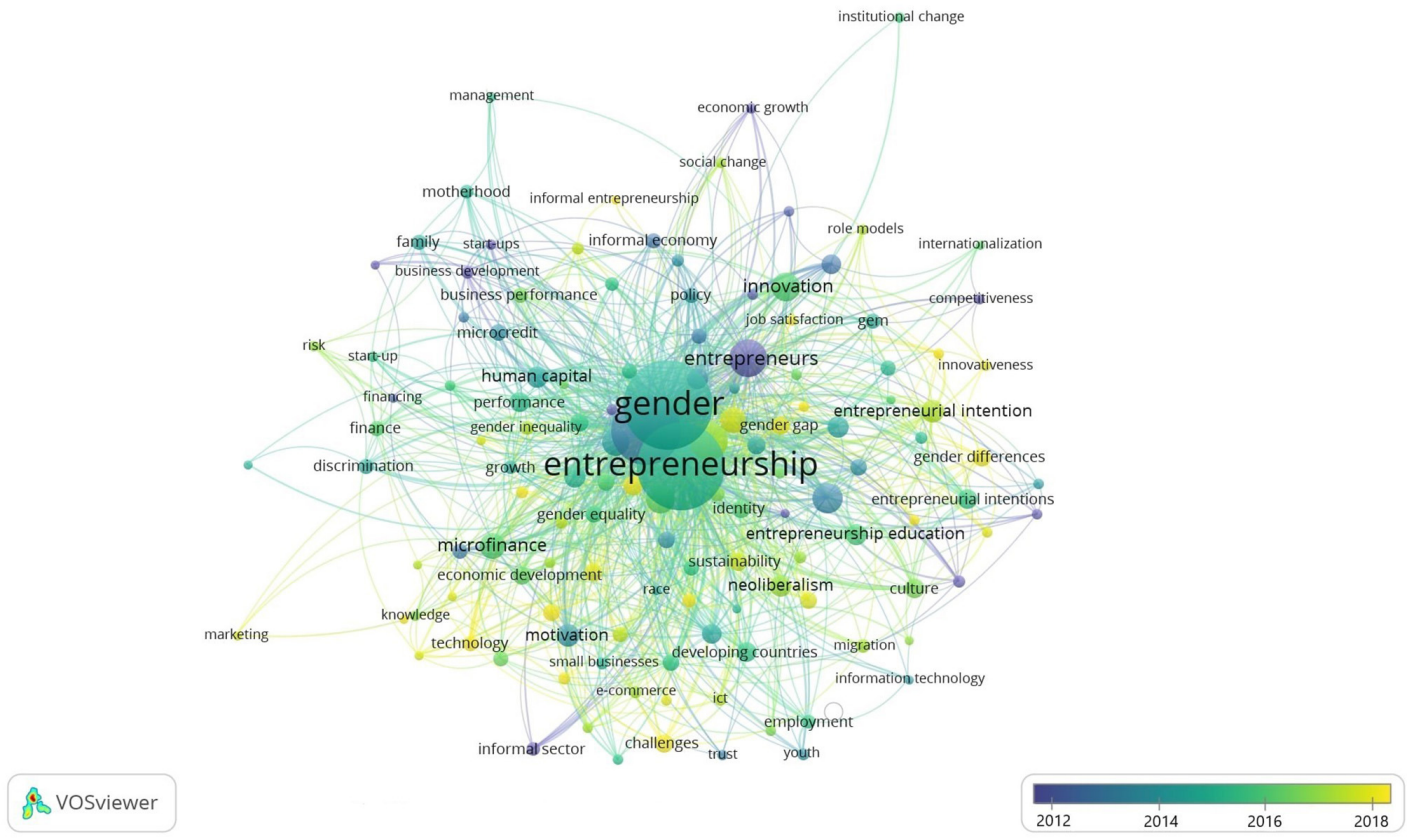
Temporal overlay on a keyword co-word occurrence map for women entrepreneurship articles.

As can be seen in Figure 3, in recent years there has been a change in interest in the international research. From observing the financing and capitalization of women's businesses (the keywords in purple: commercial development, financing, economic growth, informal economy), there has been a growing emphasis on more sensitive issues that place the need to study women's entrepreneurship as a separate field of research, with an emphasis on factors that differentiate them from its male counterpart and that allow overcoming the male-female gap in entrepreneurship (the keywords in yellow: social networks, role models, culture, entrepreneurship education, women empowerment, social entrepreneurship, family support, empowerment, social capital, self-efficacy). In fact, the relative emphasis on education, empowerment, family, social entrepreneurship, culture highlights the effort of researchers in analyzing that set of contextual and socio-psychological factors to allow the desired change.

In the 2,848 articles selected for the bibliometric analysis, a total of 3,903 authors were found, with an average of 1.95 authors per article, which shows that this is a fragmented field of research, probably due to its recent development in the scientific landscape and its multidisciplinary character. The most productive author is Marlow with 18 published articles followed by Ahl (*n* = 15 articles), Kaciak with 13 articles, Welter (*n* = 12 articles), and Orser (*n* = 10 articles).

**Figure 4:**
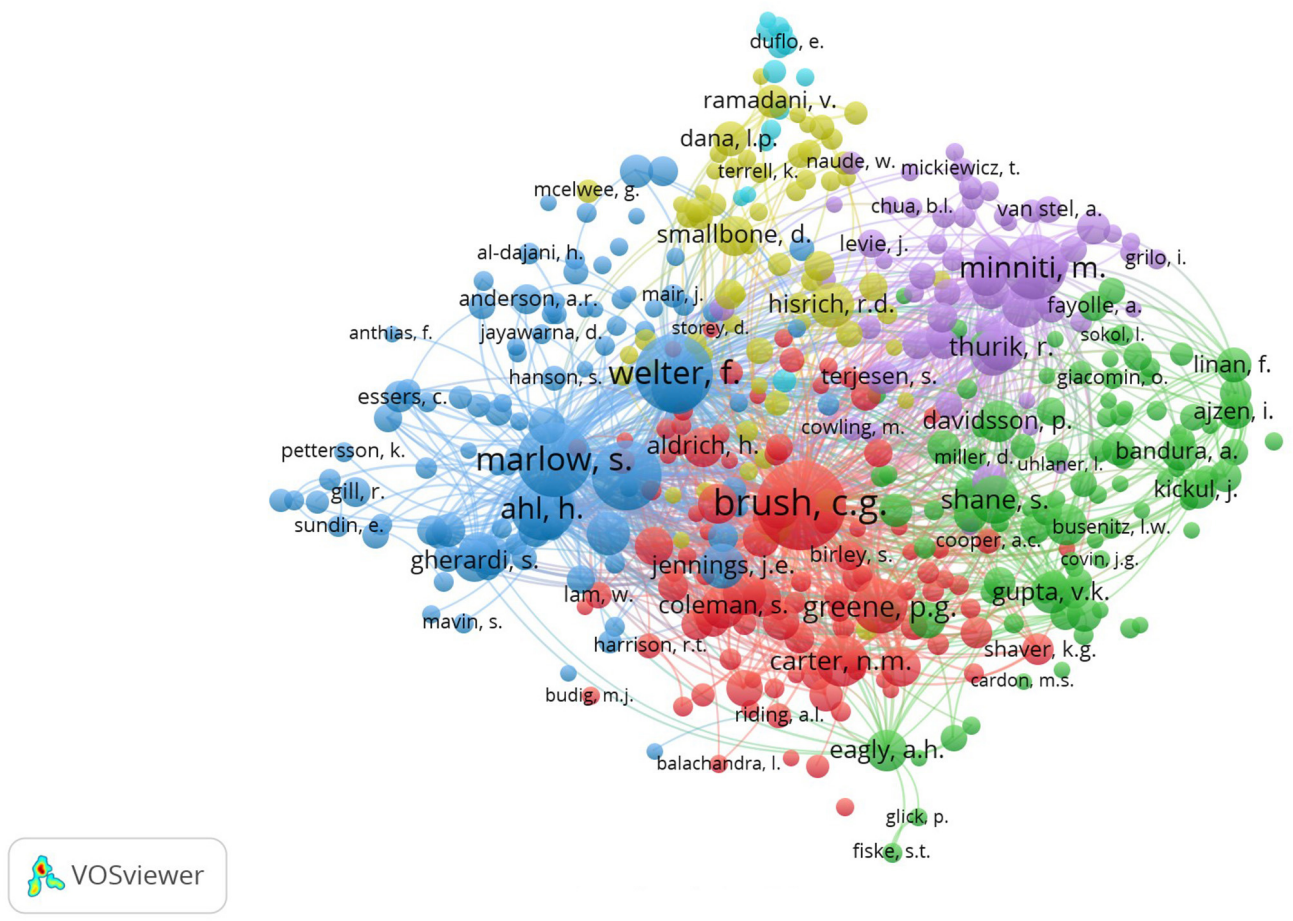
Author co-citation analysis of the woman entrepreneurship literature.

The Figure 4, indicates author co-citation analysis. Out of a total of 68,657 authors in the author co-citation network, 443 researchers met a threshold of at least 45 author co-citations.

The most highly “co-cited authors” about female entrepreneurship are Brush (1,297), Welter (992), Marlow (898), Carter (802), and Ahl (660). It should be noted that their highly cited documents tend to focus on two investigative lines of female entrepreneurship: that relating to the study of economic factors and associated barriers especially in developing countries (red cluster) and that relating to culture, gender roles and stereotypes (blue cluster). The results seem to suggest that, over the years, the interest of academics who have approached the study of female entrepreneurship has fundamentally concerned the study of barriers (economic, political, social) and the relationship between socio-cultural factors and gender-gap.

**Figure 5:**
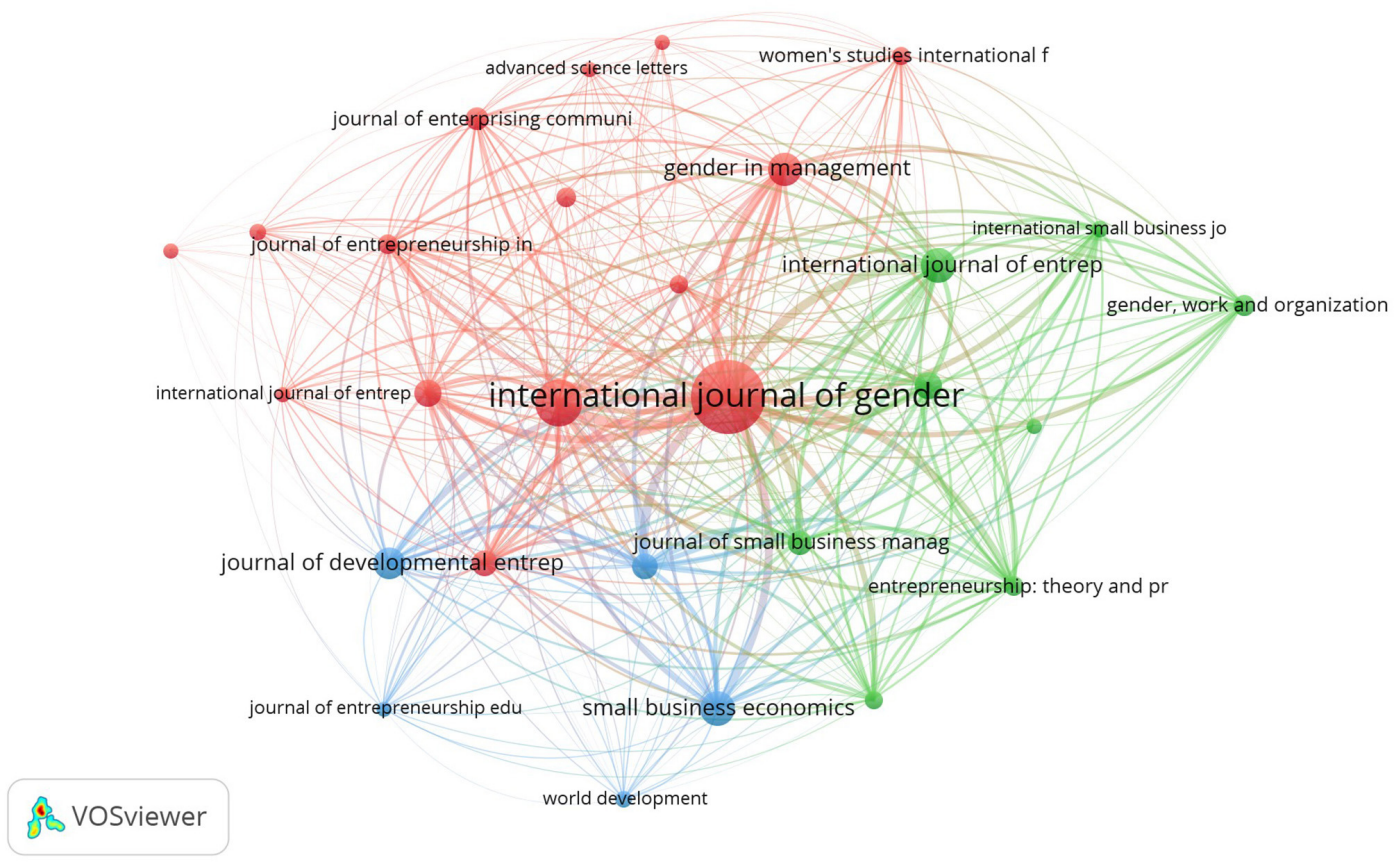
Scientific Journal analysis of the women entrepreneurship literature.

In Figure 5 we present the results of the main scientific journals that have published on female entrepreneurship. We considered the journals with at least 10 published articles, for a result of 28 scientific journals (out of a total of 841 journals). The scientific journals are displayed by circles and labels. The size of the publication circles and the label depends on the total strength of the links of a given publication. To avoid label overlap, some labels may not be visible. The color of an element is determined by the cluster to which the scientific journals belongs. The distance between two journals indicates the strength of their relationship in terms of links to common themes (Van Eck and Waltman, 2010, 2014).

These journals published 900 articles, accounting for 32.6% of the scientific production on female entrepreneurship. In particular, the scientific journals that have most published on the topic of female entrepreneurship have focused on three investigative lines: obstacles to female entrepreneurship (red cluster), the relationship between culture, gender roles and stereotypes (blue cluster) and the role of human and social capital in the growth of female enterprises (green cluster). In addition, the analysis of the research areas further clarifies the nature of the journals, underlining how these investigative lines have been treated from different perspectives (Table 2). The first 28 scientific journals, in fact, cover a differentiated range of topics such as business and management, social sciences and gender studies, human resource management; economics, law, engineering and technological innovation.

**Table 2:** Scientific journals with the most publications on the subject and Research Area.

**R**	**No. articles**	**Journals**	**TC**	**Research area**
1	151	International Journal Gender and Entrepreneurship	1329	Social Science, Gender Studies
2	82	International Journal of Entrepreneurship and Small Business	440	Business. and Manag.
3	53	Small Business Economics	727	Economics
4	48	Journal of Small Business and Entrepreneurship	398	Business and Manag.
5	47	Gender in Management	255	Gender Studies
6	46	International Journal of Entrepreneurial Behavior and Research	667	Business and Manag.
7	41	Entrepreneurship and Regional Development	527	Economics, Finance
8	37	Journal of Developmental Entrepreneurship	286	Business and Manag., Economics
9	36	International Entrepreneurship and Management Journal	290	Business and Manag., Technology Innovation
10	35	Journal of Business Venturing	408	Business and Manag.
11	31	Journal of Small Business and Enterprise Development	398	Business and Manag.
12	30	Entrepreneurship Theory and Practice	781	Business and Manag.
13	29	Journal of Small Business Management	293	Business and Manag.
14	26	Gender Work and Organization	246	Social Science, Human Resource Management
15	23	Journal of International Women's Studies	35	Social Science
16	21	Journal of Enterprising Communities	96	Business, Economics
17	19	Women's Studies International Forum	133	Social Science, Law
18	17	World Development	255	Political Science
19	15	Accademy of International Journal	13	Economics
20	15	Journal of Entrepreneurship in Emerging Economies	106	Business and Manag.
21	15	Mediterranean Journal of Social Science	31	Humanities Science
22	13	Equality Diversity and Inclusion	139	Gender Studies
23	13	International Journal of Entrepreneurship	21	Business, Social Science
24	13	Journal of Entrepreneurship Education	22	Social Science, Education
25	12	International Small Business Journal: Researching Entrepreneurship	182	Business and Manag.
26	11	Advanced Science Letters	0	Environmental Science, Health
27	11	International Journal of Innovative Technology and Exploring Engineering	1	Engineering
28	10	International Journal of Recent Technology and Engineering	0	Manag. of Technology Innovation, Engineering

*R, Rank; TC, Total Citation. Source: Elobarated by the Authors*.

This aspect to underlining the multidisciplinary nature of research about female entrepreneurship, also underlines the importance of the topic as a tool to generate value in the international economic market.

**Figure 6:**
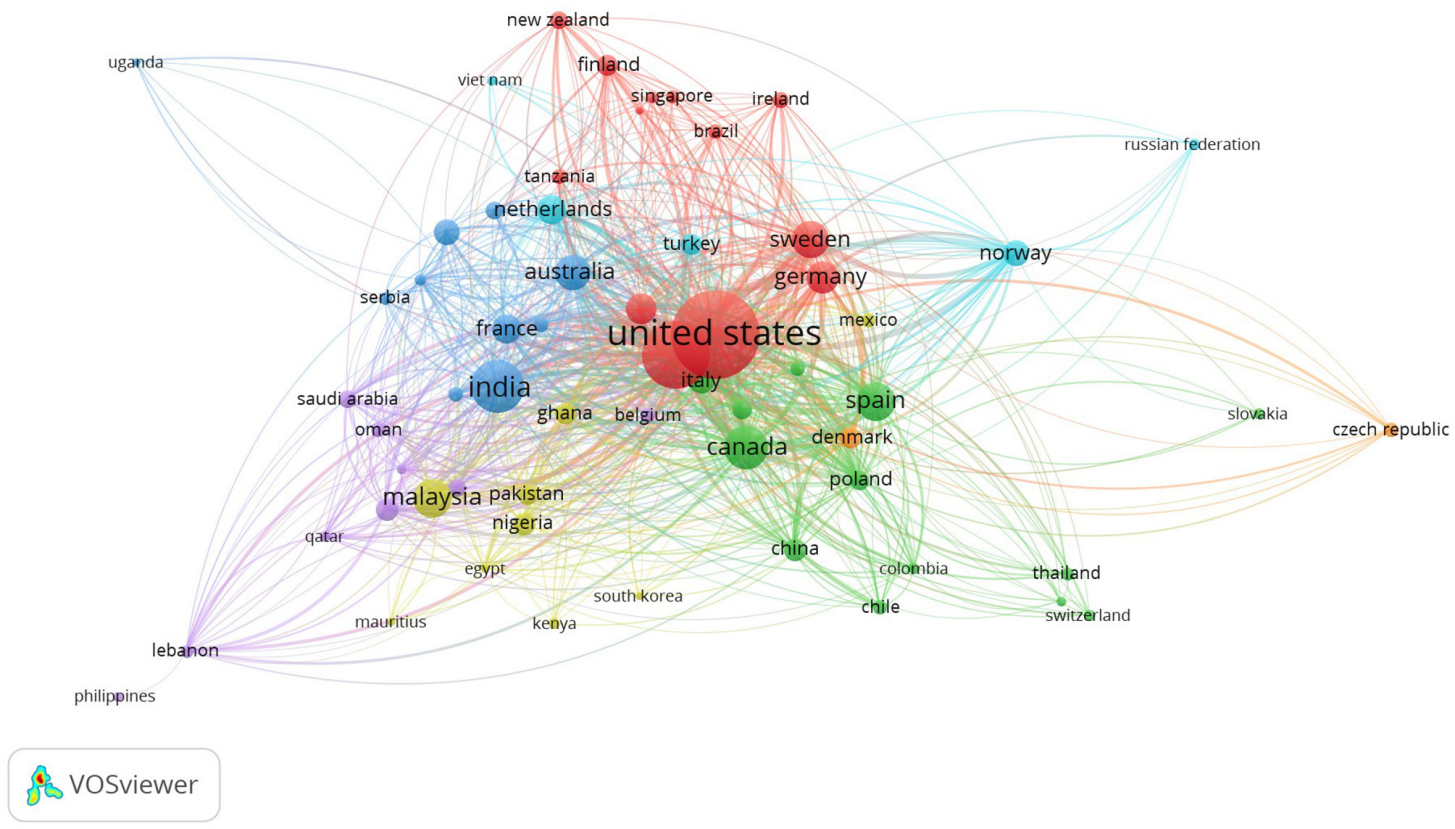
Country analysis of the women entrepreneurship literature.

With respect to the country with the most scientific contributions, the analysis showed that the United States is the nation with the greatest scientific interest, with 754 published articles, followed by the United Kingdom (*n* = 393), India (*n* = 212), Canada (*n* = 180), and Australia (*n* = 115). These five countries, mainly western countries, account for 1,654 articles (52,6%) of our full corpus of women entrepreneurship articles. Researchers in various Southern European countries (e.g., Spain: 109; Italy: 57; Portugal: 24) have also actively contributed to literature, representing a further 26% of the women entrepreneurship articles. Analyzing further, it was observed that 74% of the documents in the database came from developed companies and only 26% from developing companies (Figure 6). This result, in line with previous systematic reviews (Hallinger and Chatpinyakoop, 2019), creates a strong geographical imbalance and represents a gap in the literature that should be filled.

### Topical Clusters of the Women Entrepreneurship

**Figure 7:**
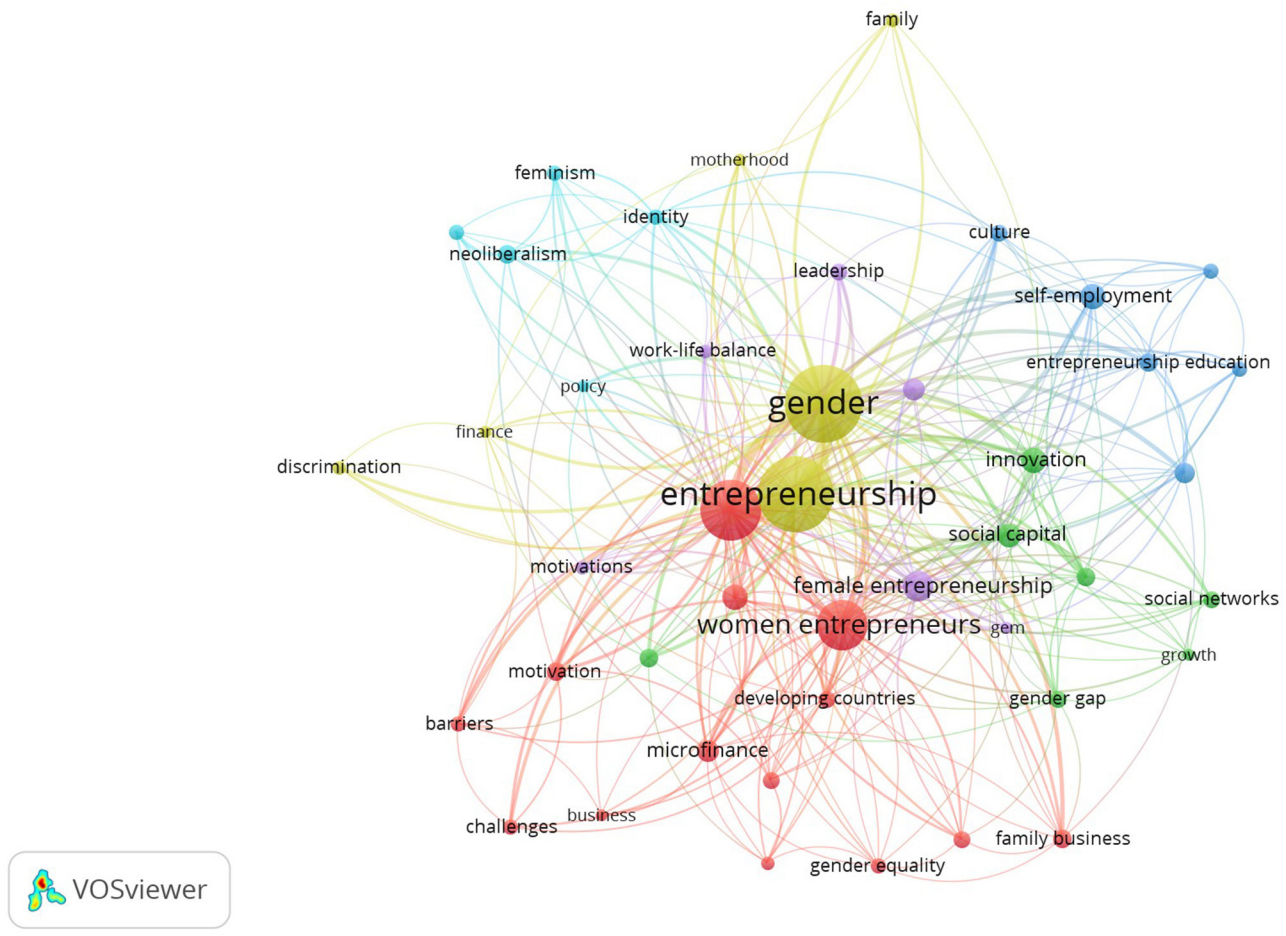
A minimum of 10 co-occurrence for author's keywords.

To get an overview of the main lines of research, we employed keyword co-occurrence analysis to reveal key topics within the women entrepreneurship knowledge base (Figure 7). In particular, with a minimum of 10 co-ocurrences per keyword and a total of 44 keywords, the topics studied most frequently by women entrepreneurship scholars cohere into six themes. It is important to keep in mind that, according to the analysis performed, the same article can be in different groups if it contains keywords that are part of several groups. The different groups are shown in Table 3.

**Table 3:** Different clusters of scientific literature.

**Cluster**	**Keywords**	**Article (out of 2,848)**	**Example of Article**
1. Barriers to women entrepreneurship 14 items	Barriers, Business, Challenges, Developing Countries, Economic Development, Empowerment, Family Business, Gender Equality, Microcredit, Micorofinance, Motivation, Performance, Women, Women Entrepreneurs	627	Al-Shami, S. S. A., Muhamad, M. R., Majid, I., and Rashid, N. (2019). Women's entrepreneurs' micro and small business performance: insights from Malaysian microcredit. *Intern. J. Entrepreneurship Small Business* 38, 312–338.
2. The role of Human and Social Capital in the growth of women enterprises 7 items	Gender Gap, Growth, Human Capital, Innovation, Small Business, Social Capital, Social Networks	394	Brush, C., Ali, A., Kelley, D., and Greene, P. (2017). The influence of human capital factors and context on women's entrepreneurship: which matters more? *J. Business Venturing Insights* 8, 105–113.
3. Culture and gender difference 6 items	Culture, Entrepreneurial Intention, Entrepreneurship Education, Gender Differences, Self-Efficacy, Self-Employment	429	Stedham, Y., and Wieland, A. (2017). Culture, benevolent and hostile sexism, and entrepreneurial intentions. *Intern. J. Entrepreneurial Behav. Res*. 23, 673–687.
4. Family support and maternity management 6 items	Discrimination, Entrepreneurship, Family, Finance, Gender, Motherhood	974	Jaafar, M., Othman, R., and Hidzir, N. I. (2015). The role of family on gender development of women construction entrepreneurs. *Adv. Environ. Biol.*, 9, 120–123.
5. Linking social entrepreneurship and women empowerment 6 items	Female Entrepreneurship, GEM, Leadership, Motivations, Social Entrepreneurship, Work-Life Balance	374	Alexandre-Leclair, L. (2017). Social entrepreneurship and social innovation as a tool of women social inclusion and sustainable heritage preservation: the case of the Sougha Establishment in UAE. *Intern. J. Entrepreneurship Small Business* 31, 345–362.
6. A feminist point of view 5 items	Feminism, Identity, Neoliberalism, Policy, Postfeminism	312	Berglund, K., Ahl, H., Pettersson, K., and Tillmar, M. (2018). Women's entrepreneurship, neoliberalism and economic justice in the postfeminist era: a discourse analysis of policy change in Sweden. *Gender Work Organ*. 25, 531–556.

*Source: Elobarated by the Authors*.

As emerged from the analysis of “front research,” in the last decade, there has been a change in interest in the international research. From observing the financing and capitalization of women's businesses, there has been a growing emphasis on more sensitive issues that place the need to study women's entrepreneurship as a separate field of research (De Carolis et al., 2009; Davis and Shaver, 2012). In this sense, in a study conducted by Dawson and Henley (2015) it was found that the gap between men and women in starting an entrepreneurial career is due to lower risk attitude expressed by women. According to Dawson and Henley (2015), the low rate of women entrepreneurs is associated with a greater fear of failure, little confidence in their skills, and perception of poor support from social networks. In addition, in a systematic analysis by Mishra (2015), the 48 articles analyzed showed that self-confidence, the provision of assistance and institutional support; and the ability to access the credit service and social networks are factors that stimulate female entrepreneurs. Similar results were found a few years earlier by Alam et al. (2011), who highlighted how personality factors (self-efficacy and risk propensity) and contextual factors (social media and professional) are intertwined. These factors, which are part of a sustainable business, are highly relevant for female entrepreneurs.

In recent years, many researchers have analyzed female entrepreneurship and its associated limitations (cluster 1, in red), especially in developing countries (Gautam and Mishra, 2016; Raghuvanshi et al., 2017). Discussing about emerging economies is extremely important, as the factors behind the low percentage of women in business activities seem to be different in developing economies than in developed economies. One could argue that women in developed countries are more likely to find suitable jobs than women in developing areas, that are also more prone to gender-related discrimination and hostile work environments (Kirby and Ibrahim, 2011; Salamzadeh et al., 2013).

Research shows that in these countries, women entrepreneurs face greater barriers (Panda, 2018; Abou-Moghli and Al-Abdallah, 2019) and that their business ventures efforts are generally discouraged (Kapinga and Montero, 2017).

For example, the systematic analysis conducted by Panda (2018) on 35 articles and 90 developing countries, reveals that the constraints faced by women stem from gender discrimination, conflict between family and work, poor access to resources, lack of training and personality differences. Specifically, they are wary of risks and suffer from isolation in their entrepreneurial path, show a lack of self-confidence and an excess of insecurity.

Raghuvanshi et al. (2017), analyzed the different barriers that female entrepreneurs face, which can be summarized as follows: lack of education, experience and training opportunities; limited spatial mobility; lack of support from families; lack of institutional support; and problem in the acquisition of financial resources. Mirghafoori et al. (2010) in his study mentioned a series of obstacles faced by women entrepreneurs in Iran that result from the lack of confidence of financial institutions toward women.

According to Okoye (2013), although in Nigeria the main need for the emancipation of women is access to financing, other problems come into play, such as the high failure rate of political support programs. Santoni and Barth (2014) concluded that the barriers faced by female entrepreneurs in developing countries are inherent due to poor access to financing and lack of institutional support. These conclusions have also been shared in the past by other studies en Irán (Galard, 2005; Sarfaraz and Faghih, 2011).

In a study conducted by Yogendrarajah and Semasinghe (2015), on a group of women from Sri Lanka, the two authors found a statistically significant relationship between the development of entrepreneurship and the microcredit program. Helping women entrepreneurs to have better access to credit means increasing their awareness in terms of risk management and self-efficacy, contributing to the family economy, improving their quality of life and, not least, reduce gender disparities.

Studies that have focused on human and social capital (cluster 2, in green) can be included in this scenario. Human and social capital resources are the key to help entrepreneurs, especially in the initial phase of their business (Brush et al., 2002). Studies in this sector have shown that high levels of human capital are positively related to the performance and management of a company (Millán et al., 2014).

The results of a study by Klyver and Schenkel (2013), based on GEM data in 41 countries, revealed that human capital is positively associated with nascent entrepreneurship and also has a positive impact on both, objective elements such as starting a business, and subjective elements such as self-perception and self-efficacy.

Studies like Aldrich and Cliff (2003) and Kirkwood (2007) have also shown that social capital is one of the biggest supporting factors for female entrepreneurship. An analysis by Ventura Fernández and Quero Gervilla (2013) shows, for example, that the existence of links with support agencies influences women's self-efficacy levels and, therefore, their intention to undertake in business activities. According to Álvarez et al. (2012), in addition to the important role of formal social capital (policy support, financing and training), it is especially the “informal capital” (family, emotional support, social network) that has the greatest impact.

However, while the relationship between these types of intangible resources and women's businesses has been widely documented in developed countries, there is limited research in emerging economies. From our analysis, we found a study that considered the impact of human, social and reputational capital on women's businesses in Ghana. The results showed the positive impact of the three types of capital on corporate growth, but, to a greater extent, they highlighted the importance of women's social networks for the growth of their businesses, and to further improve the value of their intangible skills (Sallah and Caesar, 2020).

The third theme (blue cluster) analyzes the complex relationship between culture and gender differences (Eden and Gupta, 2017), considering female entrepreneurship a result of contextual and psychological factors that differentiate it from its male counterpart.

Culture greatly influences the way in which entrepreneurs develop their business initiatives, referring to prejudices, social roles and a stereotyped vision of the gender (for example, women are seen as incompatible with the business because it is too emotional and less rational in making decisions) that contribute to a men-cenetred vision of entrepreneurship (Shinnar et al., 2012; Rubio-Bañón and Esteban-Lloret, 2016). This is reflected in Hoyt and Murphy (2016) conclusion that the prejudices women face in business are the result of gender stereotypes.

These factors related to a country's different perceptions of the role of women in society, explain that the differences concern attitudes toward entrepreneurship, but also some psychological traits that influence entrepreneurial intention: higher levels of self-efficacy, self-confidence, independence, risk appetite, and autonomy in men compared to women (Langowitz and Minniti, 2007; Robb and Watson, 2012).

In addition, women compared to the male counterpart, to a greater extent reject the choice of an entrepreneurial career because they consider themselves as lacking in entrepreneurial skills and knowledge (Wilson et al., 2007; Kirkwood, 2009) and unable to respond to the challenges of a company as it is not very socialized in corporate roles (Yordanova and Tarrazon, 2010). Ultimately, what these studies show is an issue of how gender roles could influence the types of career deemed acceptable for women, further increasing gender differences (Griffiths et al., 2013; Kalafatoglu and Mendoza, 2017).

As the cluster analysis shows, entrepreneurial education is closely linked to culture and gender differences, which is considered a potential tool for increasing entrepreneurial intentions, bridging the gap between men and women. This occurs both in the consolidation phase of the company and in the start-up phase (Mazzarol et al., 1999; Rotefoss and Kolvereid, 2005). A country that promotes entrepreneurial educational initiatives, encourages women's participation in entrepreneurship and reduces the woman-man gap (Petridou et al., 2009).

Mand et al. (2018) showed that education influences the entrepreneurship levels of Indian women even in a stereotypically masculine sector such as electronics.

Other empirical evidence has highlighted the importance of entrepreneurial education in analyzing the mediating role of self-efficacy. For example, studies have shown that entrepreneurial education has a greater impact on the development of entrepreneurial self-efficacy (Wilson et al., 2007; Centindamar et al., 2012). Others have shown that high levels of entrepreneurial self-efficacy is related to a higher probability of developing a business activity (Krueger et al., 2000; De Clercq and Arenius, 2006). Wilson et al. (2007) analyzed the role of mediating self-efficacy on the relationship between gender and entrepreneurial intention of students and adults in adulthood. In both cases, entrepreneurial self-efficacy partially mediated this relationship.

Along these factors, the literature analysis has also allowed us to identify the role of the surrounding environment, focusing mainly on the family (Cluster 4, in yellow). Our analysis showed the crucial and positive role of family members, especially when external support systems are limited (Chang et al., 2009, 2012), both as a source of economic support, especially at the start of a business (Shen et al., 2017; Cardella et al., 2020), and on the motherhood issue (Jennings and McDougald, 2007), providing moral and psychological support to women who have to reconcile family responsibilities with the desire for professional development.

A field of study in which the gap between men and women seems to be significantly reduced is that related to social entrepreneurship (cluster 5, in purple). It is an extremely recent field of research (the first article dates from 2009), and as expected, according to our analysis it is one of the clusters with the least number of publications.

In general, women seem motivated toward social goals, unlike men whose attitudes push toward more economic and material issues (Dorado and Ventresca, 2013). As the literature shows (Themudo, 2009; Hechevarría et al., 2012) social enterprises are more suited to the social role of women.

For example, Van Ryzin et al. (2009) suggests that women are more likely to be social entrepreneurs than men, since this type of company seems to share their objectives, more oriented to the attention and support of the community. In addition, according to Teasdale et al. (2011), more than 90% of women occupy management positions in voluntary organizations or the third sector. This is reflected in Kuschel and Lepeley (2016) stating that women entrepreneurs in the technology industry tend to start businesses with romantic partners, in a process of co-preneurship.

These results are in line with more recent studies that have demonstrated the importance of social entrepreneurship, especially for women who undertake in a poorly developed country (Kyalo and Kiganane, 2014; Nicolás and Rubio, 2016).

Women are motivated to choose an entrepreneurial career for different reasons than men. In general, men appreciate good pay, job security and promotion opportunities, while women prefer opportunities to use their initiative and flexible hours (Zou, 2015). The greater motivational desire among women to achieve a better balance between work and family life, leaving aside the desire for economic wealth (Thébaud, 2015), could explain the importance of social entrepreneurship as a possible career option.

For example, Muntean and Ozkazanc-Pan (2016) suggested that social enterprises can help foster reconciliation policies, such as flexible time or parental leave, which act as a motivating factor, encouraging women's career advancement.

In summary, research suggests that the intrinsic characteristics of social entrepreneurship (e.g., collaboration and mutual assistance) may be more suited to women's needs, their way of working (high quality relationships) and respect for women's priorities (like reconciliation and equal opportunities).

Additionally, as Rembulan et al. (2016) have shown, women entrepreneurs, compared to women who work as employees, have very low levels of conflict regarding time management and family care.

Lastly, cluster 6 (in light blue) includes feminist theories that attempt to explain gender discrimination in entrepreneurship as a result of stereotypes and prejudices, which deserves a discussion of its own. The articles have revealed a shift in a focus from liberal feminism, centered on a collectivist conception of women and inspired by gender equality as a political factor, to liberal post-feminism, which uses more individualistic and identity-focused vision, in which single women must compete in the national market and contribute to the economic growth of the country through self-employment (Berglund et al., 2018). In particular, the liberal feminist theory (Fischer et al., 1993), analyzed supports the need for social reform to give women the same opportunities that are reserved for men (for example, access to resources and social networks, education, previous experience in business). Liberal post-feminism, instead, takes into account many different views of the world. These differences do not imply that women are less effective in business than men, but only that they could adopt different approaches that could be as effective as the more traditional approaches adopted by men (Watson and Robinson, 2003). The problem would lie in the lack of acceptance by the social network and community, however, despite the great efforts made in this regard, both views have significant shortcomings. For example, no perspective considers the different cultural values that can convey different attitudes, expectations, and behaviors not only between men and women, but also between different nations.

Although it is widely accepted that entrepreneurship gives additional value to the economy of a nation and a shift in business, the understanding between entrepreneurship and development is still far from complete (Kelley et al., 2017). Recognizing, therefore, the factors and peculiarities that also influence the field of female entrepreneurship seems a challenge and a call to which the entire community is expected to answer at different levels. This could help academics and policy makers gain useful knowledge and facilitate the conditions of women in business. Parallel to what was stated in Holmquist and Sundin (2002), beyond the different points of view, it would be desirable to consider the issue from a holistic perspective, focusing on the strengths of each one for a vision as unitary and convincing as possible in the analysis of female entrepreneurship.

## Conclusions

The objective of this systematic analysis was to investigate the scientific literature on the relationship between entrepreneurship and women. To this end, we analyzed a total of 2,848 articles selected from the Scopus database (Scimago Research Group). Based on the results obtained, some conclusions can be drawn.

As the analysis shows, it is a relatively current area of research (the first article was published in 1950) which over the years has shown constant interest from academics, with a greater development of articles in the last 20 years.

Furthermore, it is a research field that shows a multidisciplinary character that mainly affects the area of business and management, but also social and gender studies, Economics, Political Sciences, Technology and Innovation.

In addition, in the last decade, “front research” has shown a change of interest in the scientific community, moving from the study of economic and political issues to the analysis of the useful factors that allow to bridge the male-female gap.

In general, it was possible to isolate 6 research line that characterize the current field. The topic that has received the most attention from academics and, therefore, a greater number of studies is related to the importance of the family as support for women entrepreneurs, particularly with regard to maternity management (cluster 4), closely related to cluster 1 that highlights the barriers that characterizes women's access to the entrepreneurial sector.

On the other hand, the group that according to our analysis has the least number of articles published relates to feminist theories (cluster 6), followed by cluster 5, about to the relationship between social entrepreneurship and female entrepreneurship. The latter could be explained probably because it is an extremely recent research field (the first article dates from 2009), but in constant evolution. As noted in recent studies, social entrepreneurship is a very interesting field of analysis, since the gap between women and men is greatly reduced because the roles and stereotypes that influence women's behavior lead to identify better with the values present in social enterprises (Nicolás and Rubio, 2016).

Although this work covers a large number of publications, some limitations must be pointed out. First, it may be important to use other databases to expand the body of literature and highlight the differences and similarities with the analysis presented by us. It may also be of interest to use different bibliometric indicators to continue studying the research fields.

For this study, we used cluster analysis to delineate the boundaries of scientific literature through VosViewer 1.6.10 software. It is a tool that, although it has received a broad consensus from researchers (Martínez-López et al., 2018), presents some limitations, since it provides a limited number of relationships that, based on similarities and co-occurrence techniques, only take into account the frequency of the keywords considered. This could represent a limitation if the search field is excessively fragmented. For example, a total of 4,455 keywords emerged from our analysis, and the field was reduced to 44 keywords, establishing a co-ocurrence of 10. This indicates that it is an extremely varied field of study in which academics have adopted different points of view.

From this study, some suggestions for future research can also be outlined.

First, the vast majority of women's business studies have been carried out in western and developed countries. It would be appropriate for academics to deepen this issue in developing areas in order to test the theories already used, analyze the dynamics that are created in these different geographical areas, as well shed light on the social and cultural challenges women face in these contexts. Additionally, this aspect was also recently confirmed by Rashid and Ratten (2020), who in their systematic review of female entrepreneurship in emerging economies, found only a total of 76 published articles.

Future studies should also reflect on the fact that more and more women participate in the growth of their businesses, going beyond the initial phase, which concerns simple entrepreneurial intention. The articles that are part of this systematic analysis did not take into account the difference between intention and behavior. It would be advisable to focus more on this difference, emphasizing whether women who are in a later stage face the same specific challenges as in the early stages. Entrepreneurship is more than the simple act of starting a business, since it also represents the will and desire to manage an existing company (see the case of family businesses). Therefore, it would be desirable, especially with reference to women, to reflect on the barriers they face to grow their businesses, in terms of work/family balance issues, choice of professional sector and identification of opportunities and development of human and social capital. In this sense, a great importance for women derives not only from access to financial resources, but also from intangible resources (human and social capital) that constitute the key to business success in general, and specifically in women. Studies in this area, however, seem limited. A careful reflection on women entrepreneurs, both in developed and developing economies, could help to better understand how to exploit these resources.

Furthermore, it could be a further reason for reflection, analyzing the problem of immigrant women entrepreneurs, the motivations that push them to start entrepreneurial activities, the social consequences of their entrepreneurial behavior and how the whole process is conditioned by their belonging to the female gender.

From a purely methodological point of view, we expect the use of diversified, quantitative, qualitative or mixed approaches, since this may offer greater potential to analyze different nuances and peculiarities that may be important to deepen on the female issues in entrepreneurship. Similarly, the increasing availability of large data sets allows us to understand possible disadvantages among different groups of female entrepreneurs (Fairlie and Robb, 2008). The comparisons between different female groups in social, cultural and socio-family abstractions, with difficult access to human and intangible resources and financial resources, remain important fields of analysis and exploration.

Finally, there is a need for greater efforts by academics to critically reflect and strengthen current theories on entrepreneurship, which should be useful for the development of more solidified theories that take into account culture and institutional practices and how they relate with gender issues (Wilson and Tagg, 2010).

We believe that the results of this systematic analysis are a starting point for contributing to an ever clearer systematization of the scientific literature, which, given the very varied nature of the research topic, has some limits which are not always easy to define. In our analysis, we have adopted a holistic point of view to give voice to the different theoretical contributions that have tried to explain the many facets of the research lines. The synthesis of topics of recent interest among scholars has produced numerous topical clusters and a change of interest, over the years, from a study aimed at economic issues to an analysis that deepens the factors that contribute to reducing woman-man gap.

We interpret it in the sense that scholars have started to take an interest in female entrepreneurship as an independent construct and not simply as a counterpart to the male one, applying already existing models concerning male entrepreneurship. This also represents a useful starting point for political systems and further strengthens our conclusions.

In light of the results found in our study, we can affirm that the work done gives us the opportunity to have a broader vision of gender and women's entrepreneurship, not only considering the motivations, objectives, measures of success and the different contexts, in which their ventures are managed and developed, but also taking into account the heterogeneity of female entrepreneurs in general.

The researchers recognize that women's entrepreneurship is very varied and rich in nuances, hence the commitment that must exist in researchers to commit to this complexity and, at the same time, strength. Women entrepreneurs are not a homogeneous group, and therefore we must recognize gender identities, which are rarely considered in the entrepreneurship literature. This is especially important when it comes to, for example, programs and policies to support female entrepreneurship. We cannot do “one size fits all” training. In other words, female entrepreneurs are not minors, subordinates, they simply do entrepreneurship differently and in this process make significant and valuable contributions to the global economy. A better understanding of this diversity of female entrepreneurship will surely contribute new ideas for research on entrepreneurship in general.

Our research also opens up new questions that will need to be addressed in future research. For example: (a) How do different contexts (organizational, family environments, high technology, etc.) and cultural environments impact on women's business management? How do women entrepreneurs undertake in such contexts? (b) How can theories outside of entrepreneurship/small business fields shed light on women's entrepreneurship and its management strategies? (c) How should women's entrepreneurship best be conceptualized to better understand the diversity, strategic management and growth dimensions of business creation? With new theoretical and methodological approaches and perspectives, we can address these questions.

## Data Availability Statement

All datasets generated for this study are included in the article/supplementary material.

## Author Contributions

All three authors participated in the analysis and drafting of this document. Specifically, GC has selected and analyzed all the articles present in Scopus. BH-S provided interesting details on the subject. JS-G examined the methodology used and the final draft of the document. The authors decided to approve the final work and take full responsibility for the originality of the research.

## Conflict of Interest

The authors declare that the research was conducted in the absence of any commercial or financial relationships that could be construed as a potential conflict of interest.
